# Physiological and agronomical evaluation of elite rice varieties for adaptation to heat stress

**DOI:** 10.1186/s12870-022-03604-x

**Published:** 2022-05-10

**Authors:** Vincent Ezin, Wassiou Worou Ahanchede, Mathieu Anatole Tele Ayenan, Adam Ahanchede

**Affiliations:** 1 Department of Crop Production, Faculty of Agricultural Sciences, University of Abomey-Calavi, 01 BP 526, Cotonou, Benin; 2 West Africa Centre for Crop Improvement, College of Basic and Applied Science, University of Ghana, Legon, Ghana; 3 World Vegetable Center, West and Central Africa-Coastal and Humid Regions, IITA-Benin Campus, Cotonou, Benin

**Keywords:** Rice varieties, Heat stress, Heritability, Genetic diversity, Benin

## Abstract

**Background:**

The increasing temperatures due to climate change around the world poses a serious threat to sustainable crop production. The growing adverse effects of heat stress are putting global food security at great risk. Crop improvement for adaptation to increased temperatures is therefore of paramount importance. This study aims at assessing the effects of heat stress in relation to agro-morphological and physiological traits of six rice varieties. The study was carried out in the Township of Glazoué, a rice-growing area in Benin. The experiments were laid in randomized complete block design with three replications. Two types of stress were imposed: high-temperature stress in the dry season and optimal temperatures in the rainy season. The calculated mean values of morphological, physiological, and agronomic traits were used to estimate heritability, genetic advance, PCA, and correlation.

**Results:**

The results showed that heat stress had a significant (*p* ≤ 0.01) influence on plant height, leaf length, number of tillers, number of internodes, days to flowering, and days to maturity, 1000-seed weight, and yield per plant. The heat stress had significantly delayed the flowering of all the varieties when compared to the controls. The highest values of 1000-seed weight (34. 67 g) were recorded for BRIZ-8B while the lowest (25.33 g) were recorded for NERICA-L20. The highest values for the genotypic coefficient of variation (43.05%) and phenotypic coefficient of variation (99.13%) were recorded for yield per plant under heat stress. The topmost broad-sense heritability was recorded for grain width (92.72%), followed by days to maturity (69.33%), days to flowering (68.50%), number of grains per panicle (57.35%), and yield (54.55%).

**Conclusions:**

These results showed that BRIZ-8B and BRIZ-10B were the most tolerant to high temperature amongst the six varieties assessed and potentially could be recommended to farmers for production under high temperature and be used in breeding programs to improve heat tolerance in rice.

**Supplementary Information:**

The online version contains supplementary material available at 10.1186/s12870-022-03604-x.

## Background

Rice is among the most consumed staple foods in the world with a total production of 778.6 million tons in 2018 [1]. Globally, the trend of rice consumption is sharply increasing in parallel with global population growth, especially in Africa and Asia where a large proportion of the population generally rely on rice as a daily source of calories [2]. Thus, massive intensification of rice cultivation is needed to ensure global food security. In Africa, rice has become a strategic food because of its contribution to household food security and its impact on national economies [3]. Like most African countries, Benin is faced with changing dietary habits characterized by the increase in rice consumption, which was once considered as a festive food [4]. Consequently, the consumption needs of rice have significantly increased and the national demand for rice was evaluated at 376560 tons in 2015 [5]. Despite the increase in national production from 200,000 tons in 2013 to 459,313 tons in 2018 [1], the production still does not meet the growing demand of the population. The increase in production is related to both yield and harvested area with an annual production growth rate of 3.06 and 8.37%, in 2013 and 2018, respectively [1].

Benin has a large amount of cultivable land for rice production with 205,000 ha of available wetlands [6]. However, it should be noted that the average yield (3 t/ha) is still lower than the world average yield (4 t/ha) [7]. The upshot of all this is that not only the yield is low, but also most of the available lands are not used for rice production. Several organizational, biotic, and abiotic constraints underly this observation. Most of the abiotic stresses are related to soil salinity [8, 9] drought [10], and heat stress [11]. Also, the result of surveys conducted by the AfricaRice in the 12 sub-Saharan African countries showed that drought and climate change effects cause yield losses up to 33% [12].

Rice production has also been intensified in lowland and dryland (upland) rained cropping systems, many of which are threatened by drought and high temperatures [13]. In addition, global climate change is likely to aggravate the current vulnerability of cropping to climate, with a projected increase in global average surface temperature of 1.4–5.8 °C by 2100 and the potential for increased variability in this average [14]. High temperature is generally referred to as an increase in temperature above a threshold for a spell that causes irremediable destruction to crops during growth and development [15]. Temperature increase caused by climate variation affects rice production. Previous studies have shown that the flowering stage and to a lesser extent grain filling are the most susceptible developmental stages of rice to high temperatures [16–18]. When rice is subjected to air temperatures above 35 °C, heat injury occurs and two developmental stages are distinguished as the most sensitive to high temperatures; these include the time of flowering and the interval about 9 days before flowering [19]. The projected temperature increase of 2–4 °C by the end of the 21^e^ century [20], poses a threat to rice production. Rice yields under these conditions will be very low, mostly below average, because farmers continue to grow heat sensitive varieties; this is justified by the non-existence of modern high-yielding varieties that are tolerant to heat stress. Controlling high temperatures is a real bottleneck in rice production in Benin. Breeding for heat tolerance has been identified as a cost-efficient option to cope with heat stress [17, 21]. This requires prior knowledge on the heat tolerance status of available germplasm collection. However, in Benin, no information is available on heat-stress-tolerant rice varieties that could be used under local conditions.

This study aims at screening and identifying heat tolerant rice varieties in Benin based on agro-morphological and physiological parameters.

## Materials and methods

### Study area

**Fig. 1:**
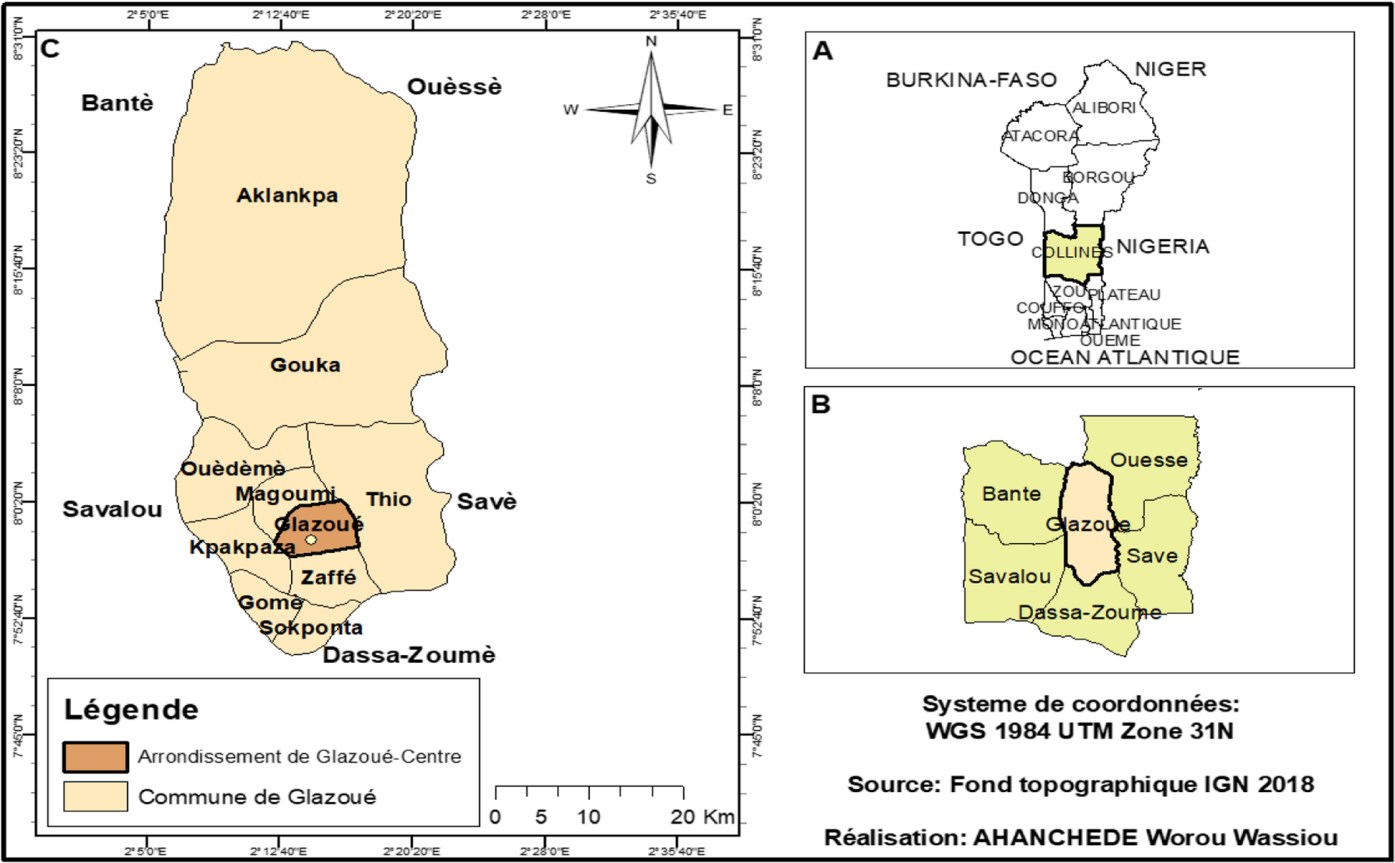
Location of the experimental site

The study was carried out in the Township of Glazoué, located between 2°14′ East longitude and 7°58′ north. The average annual rainfall ranged from 959.56 mm to 1255.5 mm with a peak in July. Figure 1 shows the Township of Glazoué and the location of the experimental site.

#### Plant material

A total of six varieties from the cultivated rice species *Oryza sativa* (IR841) and 05 interspecific varieties (BRIZ-8B, BRIZ-9B, BRIZ-10B) constituted the plant material used (Table 1). They were obtained from the National Agricultural Research Institute of Benin (INRAB). The collection of the rice varieties used in this study complied with institutional, national and international guidelines and legislation.

**Table 1:** Characteristics of plant materials used in this study

Varieties	Pedigree	Parent	Status	Ecology
IR841	WAS-122	TOG5681	*O. sativa*	Lowland
NERICA-L14	–	–	Interspecific	Lowland
NERICA-L20	WAS-122-IDSA-WAS-1-WAS-B	TOG5681/3*IR64	Interspecific	Lowland
BRIZ-8B	WAS6122-IDSA-1-WAS-6-1	TOG5681/3*IR64	Interspecific	Lowland
BRIZ-9B	WAS-191-8-3	IR64/TOG5681//4*IR64	Interspecific	Lowland
BRIZ-10B	–	–	Interspecific	Lowland

#### Experimental design

The study was conducted in a randomized complete block design with 3 replicates and six varieties. Two experiments were conducted. The first experiment was carried out from February to June 2020, the periods of high temperatures in the study area but it should be noted that after flowering the rains began and the temperatures became optimal, which favored the plant recovery and productivity. The second experiment, considered as a control, was conducted from July to November 2020, which is the optimal rice growing season in the study area. Two factors, namely varieties and temperatures were studied.

#### Plant management

The seeds were sown and raised in plastic trays for 21 days, and the seedlings were transplanted in rows with two plants per hole according to the pre-established randomization plan. Each variety was planted in 7 rows per plot with an inter-row and intra-row spacing of 25 cm. The data were collected from plants on the 5 rows in the middle while the 2 other rows served as border rows. At vegetative stage, fertilizers were applied at the rate of 100 kg/ha for NPK and 50 kg/ha for Urea. Manual weeding was done to keep the field free of weeds.

### Data collection

Daily temperature data of the experimental sites were recorded during the two trials. The growth parameters measured were: plant height, plant diameter at the base point of the stem or tiller, leaf length, and width, number of tillers, number of internodes, internode length, panicle leaf length and width, and plant vigor. These measurements were made on 9 plants per plot. The yield parameters measured included length and width of rice grains, panicle length, number of primary and secondary branches, the average number of empty and full grains per panicle, and weight of 1000 rice grains. Yield-related data were measured on ten selected panicles per plot at harvest.

Physiological data were collected using chlorophyll fluorescence meter (OS30p Opti-Sciences) from fully expanded leaflets clipped from the upper part of the plant canopy, after a 1-h dark adaptation period. Dark fluorescence (Fo), maximal fluorescence (Fm), and photochemical yield (Fv / Fm, where Fv = Fm - Fo) were recorded.

### Data analysis

The analysis of variance (ANOVA) was carried out for all the quantitative data to check whether the factors or their interaction had a significant effect on the parameters. The analysis was performed using R version 4.0.3 (R Core Team, 2021). The means under optimal and high temperatures were compared using Boxplots. The significance level for statistical analysis was set at 5%. The results of the analysis of variance were used to estimate the variance component to calculate the genetic parameters (Table 2).

**Table 2:** Formulas for the different genetic parameters estimated

Genetic Parameters	Formulas	Meaning of terms
Genotypic Variance (GV) Phenotypic Variance (PV) Heritability in the broad sense (H^2^)	VG = (MSG-MSE)/r VP=VG+(MSE)/r = MSG/r H^2^ = (VG/VP)*100	MSG: mean square of the genotype MSE: mean square of the error r: number of replications
Coefficient of genotypic variation (GCV) Coefficient of phenotypic variation (PCV)	GCV(%) = ($\sqrt{VG}/$ X)*100 PCV(%) = ($\sqrt{VP}/$ X)*100	√VG = standard deviation of genotypic variance √VP = standard deviation of the phenotypic variance X = trait mean

The Principal component analysis (PCA) was used to study the correlations between the variables of growth and physiological parameters, and yields and yield components on the different axes which summarized more than half of the information, PCA was also carried out to identify the main features contributing to the variability observed between the varieties. The descriptive statistics highlighting the means of the homogeneous classes served to determine the traits of discrimination of the different groups of varieties generated by the dendrogram. The results of the analysis of variance made it possible to calculate the genetic parameters (Table 2).

## Results

### Variation in temperature during the trials

**Fig. 2:**
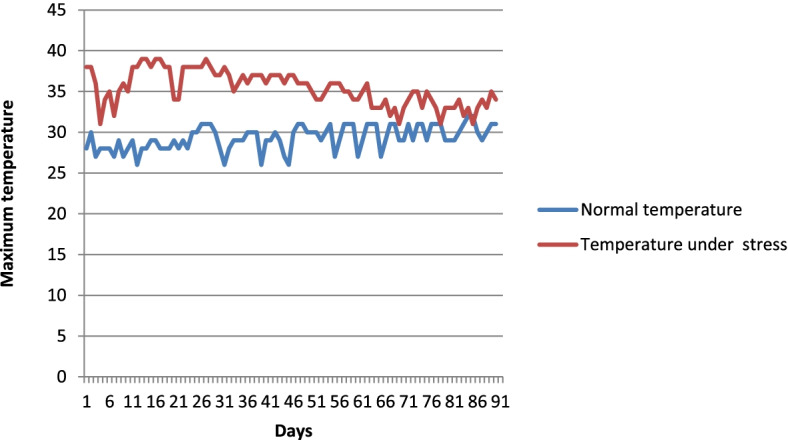
Temperature variation in normal condition and under heat stress

The maximum temperatures recorded for 90 days during both trials using the Google Weather application are presented in Fig. 2 and Supplementary Table. During the heat stress season, the temperatures ranged from 36 °C to 39 °C degrees. The highest daily maximum temperature under heat stress condition (39 °C) was recorded in March during the tillering stage The average maximum temperatures under heat stress during the flowering stage was 35 °C under heat stress conditions. The average relative humidity during heat stress was 40%.

### Effect of temperature on growth traits

Analysis of the variance (Table 3) revealed that the variety had a significant effect on plant height and leaf length at 30 days after transplanting and on leaf length at 56 days after transplanting (*P* ≤ 0.0001), panicle leaf length (*P* ≤ 0.001), plant height at 56 days after transplanting and panicle leaf width (*P* ≤ 0.01).

**Table 3:** Significance of plant growth variables

	*F*-value and Probability		
	Variety	Temperature	Variety^a^Temperature
HP30	26.10^c^	19.46^c^	3.074^a^
HP56	3.44a	11.72^b^	1.38 ns
Diat30	1.97 ns	57.44^c^	1.49 ns
Diat56	0.44 ns	0.34 ns	0.63 ns
LongF30	7.53^c^	1.21 ns	1.40 ns
LongF56	19.28^c^	7.46^a^	3.075^a^
NbT30	2.12 ns	48.37^c^	0.70 ns
NbT56	1.99 ns	46.59^c^	1.21 ns
NbEN30	0.66 ns	10.66^c^	0.66 ns
NbEN56	1.15 ns	0.09 ns	1.13 ns
LongEN30	2.26 ns	44.10^c^	3.06^a^
LongEN56	0.82 ns	18.24^c^	0.924 ns
LongFP	5.04^b^	0.00 ns	0.11 ns
LargFP	2.20^a^	9.09^b^	0.50 ns

Significant (^a^); highly significant (^b^); very highly significant (^c^) *HP30 *plant height at 30 days after transplanting (in cm), *HP56 *height at 56 days after transplanting (in cm), *Diat30 *diameter at 30 days after transplanting (in mm), *Diat56 *diameter at 56 days after transplanting (in mm), *LongF30 *leaf length at 30 days after transplanting (in cm), *LongF56 *leaf length at 56 days after transplanting (in cm), *NbT30 *number of tillers at 30 days after transplanting,  *NbT56 *number of tillers at 56 days after transplanting, *NbEN30 *number of internodes at 30 days after transplanting, *NbEN56 *number of internodes at 56 days after transplanting, *LongEN30* internode length at 30 days after transplanting (in cm), *LongEN56 *internode length at 56 days after transplanting (in cm), *LongFP *panicle leaf length (in cm), *LargFP* panicle leaf width (in cm)

Temperature had significant influence on plant height and plant diameter at the base point of tiller at 30 days after transplanting, number of tillers at 30 and 56 days after transplanting, number of internodes at 30 days after transplanting, internode length at 30 and 56 days after transplanting, and plant vigor at 30 days after transplanting (*P* ≤ 0.0001), plant height at 56 days after transplanting and panicle leaf width (*P* ≤ 0.001) (Table 3).

The temperature by variety interaction had a significant effect (*P* < 0.01) on plant height at 30 days, leaf length 56 days after transplanting, and internode length measured at 30 days after transplanting (Table 3).

### Effect of temperature on yield traits

The analysis of the variance (Table 4) showed that the variety had a very highly significant effect (*P* ≤ 0.0001) on the days to 1% flowering, the days to 50% flowering, the days to maturity of grains, the width of grains, the weight of 1000 grains and the average yield per plant. We observed a significant effect (*P* ≤ 0.01) of variety on length of panicles, average number of filled grains and the yield per plant (Table 4).

**Table 4:** Analysis of variance of yield and yield components

	*F* value and Probability		
	Variety	Temperature	Variety^a^ Temperature
NPa	2.49	1.46 ns	0.39 ns
LongPa	3.87^a^	0.34 ns	0.27 ns
DatF1%	7.46^b^	11.78^b^	0.69 ns
DatF50%	29.67^b^	47.96^b^	3.807^a^
DatM	11.73^b^	47.96^b^	3.80^a^
LongG	1 ns	1 ns	1 ns
LargG	8.20^b^	1.0 ns	0.2 ns
NbRFP	1.65 ns	1.12 ns	1.07 ns
NbRFS	2.118	207.409^b^	3.255^a^
NbMGP	2.73^a^	1.25 ns	1.34 ns
NbMGV	2.58	0.12 ns	0.07 ns
P1000G	37.62^b^	36.76^b^	2.939^a^
RdmPlt	6.56^b^	0.80 ns	1.28 ns
RdPa	3.626^a^	0.0001 ns	0.85 ns

Significant (^a^); very highly significant (^b^), *NPa *number of Panicle, *LongPa *panicle length, *DatF1% *days to 1% flowering, *DatF50% *days to 50% flowering, *DatM *days to grain maturity, *LongG *grain length (in mm), *LargG *grain width (in mm), *NbRFP *number of primary branches, *NbRFS *number of secondary ramification, *NbMGP *average number of filled grains per panicle, *NbMGV *average number of empty grains per panicle, *P1000G* weight of thousand grains, *RdmPlt *average yield per rice plant (in kg/m2), *RdPa *yield per plant (in t/ha)

The analysis of variance (Table 4) showed that the temperature had a significant effect (*P* ≤ 0.0001) on days to 1% flowering, the days to 50% flowering, the days to maturity of grains, number of secondary branches, and the weight of 1000G.

Variety by temperature interaction had a significant influence (*P* ≤ 0.01) on the days to 50% flowering date, days to grain maturity, number of secondary branches number, and 1000G weight.

### Effect of temperature on physiological traits

The analysis of variance (Table 5) showed that variety had a significant influence only on the initial value of fluorescence taken at 30 days after transplanting.

**Table 5:** Significance of physiological variables

SSV	F value and Probability		
	Variety	Temperature	Variety^a ^Temperature
Fo30	3.62a	0.0001 ns	0.85 ns
Fo40	0.43 ns	3.26^a^	1.12 ns
Fv30	0.40 ns	0.0002 ns	0.92 ns
Fv40	0.53 ns	6.24^a^	1.49 ns
Fm30	0.42 ns	0.025 ns	0.82 ns
Fm40	0.27 ns	2.72 ns	1.87 ns
(Fv/Fm) 30	1 ns	1 ns	1 ns
(Fv/Fm) 40	1 ns	1 ns	1 ns
(Fv/Fo) 30	0.34 ns	0.90 ns	0.66 ns
(Fv/Fo) 40	0.99 ns	12.19^b^	0.99 ns

Significant (^a^); highly significant (^b^); *Fo30 *Initial value of fluorescence after 30 days of transplanting, *Fo40 *Initial value of fluorescence after 40 days of transplanting, *Fm30 *Maximum value of fluorescence after 30 days of transplanting, *Fm40 *Maximum value of fluorescence after 40 days of transplanting, *Fv30* Fluorescence to variable taken at 30 days after transplanting, *(Fv/F0)30 *Ratio between variable and initial fluorescence taken at 30 days after transplanting, *(Fv/F0)40 *Ratio between variable and initial fluorescence taken at 40 days after transplanting, *(Fv/Fm) 30 *Ratio between variable and maximum fluorescence taken at 30 days after transplanting

Similarly, temperature had a highly significant influence (*P* ≤ 0.001) on the ratio of variable to initial fluorescence (Fv/Fo) taken at 40 days after transplanting. Temperature had a significant effect (*P* ≤ 0.01) on the initial fluorescence value (Fo) at 30 days after transplanting and the variable fluorescence (Fv) at 40 days after transplanting. In contrast, temperature had no effect on the rest of the physiological parameters.

The interaction between variety and temperature had no effect on the physiological traits. It is therefore concluded that no physiological variable explained the influence of temperature on the varieties.

### Comparison of varieties under the two environmental conditions

**Fig. 3:**
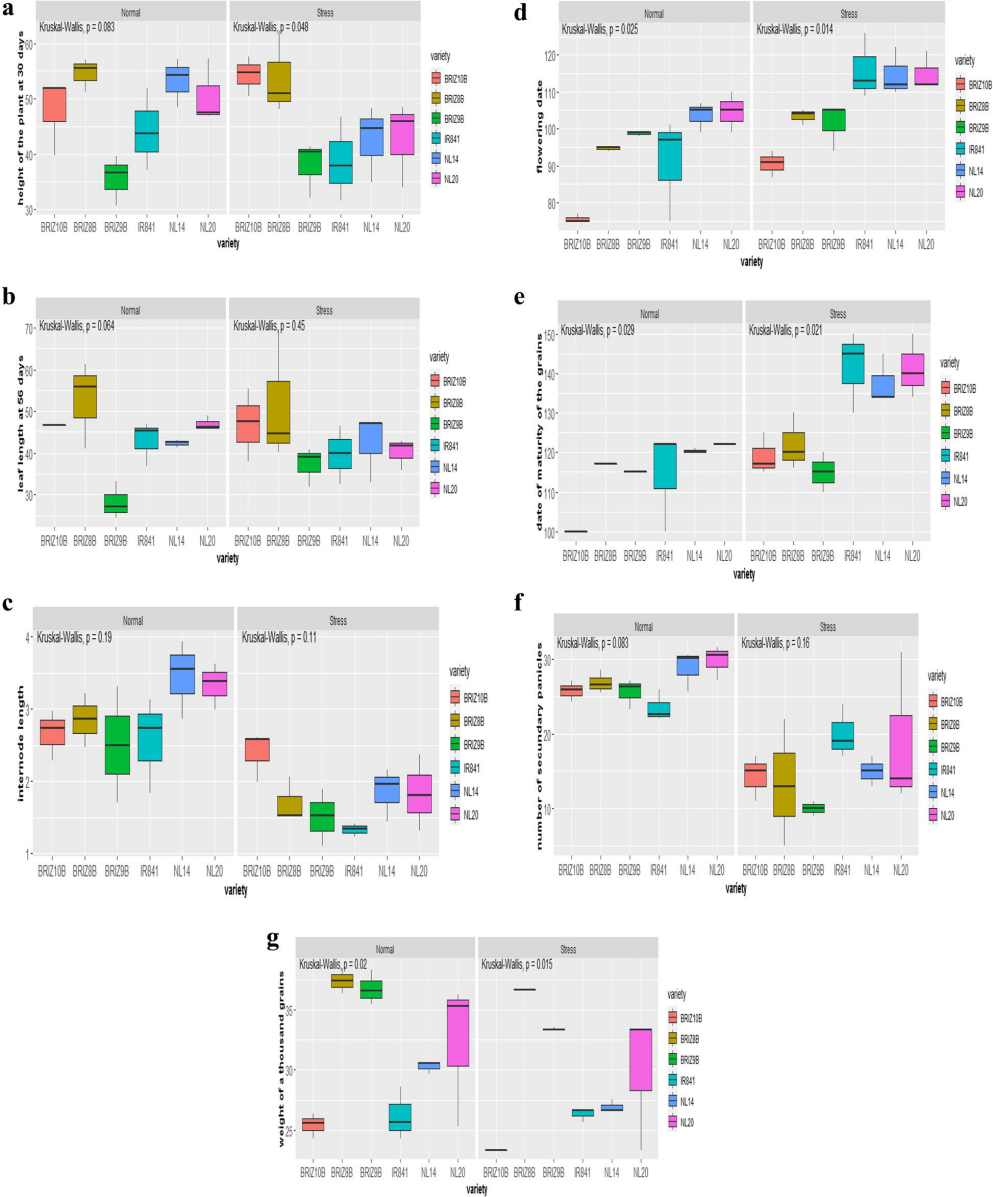
**a** Boxplots of plant height under normal condition (left) and heat stress (right). **b** Boxplots of leaf length under normal condition (left) and heat stress (right). **c** Boxplots of internode length under normal condition (left) and heat stress (right). **d** Boxplots of flowering date under normal condition (left) and heat stress (right). **e** Boxplots of the maturity grain date under normal condition (left) and heat stress (right). **f** Boxplots of the number of secondary panicles. **g** Boxplots of the weight of thousand grains

The variety with the highest plant height under normal conditions (73.96 cm) and heat stress (60.80 cm) was BRIZ-8B (Fig. 3a). BRIZ-9B had the shortest plant height under both optimal and heat stress conditions.

Under optimal conditions and heat stress, BRIZ-8B recorded the longest leaves at 56 days after transplanting (Fig. 3b).

Under normal temperature regimes and heat stress, NERICA-L14 and NERICA-L20 had the highest averages of internode length at 30 days (Fig. 3c).

Under optimal conditions, NERICA-L20, NERICA-L14, IR841, BERIZ-9B, and BRIZ-8B had the longest days from sowing date to 50% flowering with an average of 98 days, 97 days, 97 days, and 94 days, respectively. While under heat stress, IR841, NERICA-L20 and NERICA- L14 flowered late (115 days, 115 days, 114 days, respectively). BERIZ-10B had the shortest number of days to 50% flowering under both optimal and heat stress conditions.

Under heat stress, it is observed that NERICA-L20, NERICA-L14, and IR841 matured late at 142 days, 141 days, and 137 days, respectively (Fig. 3e). Under optimal and heat stress conditions, BRIZ-9B showed the shortest days to grain maturity (100 days) (Fig. 3e). Regardless of the conditions, IR841 had the highest variability in days to maturity. Overall, the varieties exhibited higher variation and took longer to mature under heat stress compared to normal conditions (Fig. 3e).

Under optimal conditions, NERICA-L20 had the highest number of secondary branches (30.00), followed by NERICA-L14 (29.00) and BERIZ-8B (27.33). The variety IR841 had the lowest number of secondary branches under optimal conditions with an average of 23.66; while under heat stress the variety BERIZ-8B recorded a low number of secondary branches (12.00).

Under optimal temperature and heat stress conditions, two varieties BERIZ-8B and BERIZ-9B had the highest weight of 1000 grains. Under optimal conditions, BERIZ-8B and BERIZ-9B recorded 37.44 g and 36.78 g, respectively while under heat stress, they recorded 34.67 g and 32.41 g, respectively.

### The hierarchical ascending classification (HAC) of rice varieties under both temperature conditions

HAC was used to assess the similarity and dissimilarity between the varieties based on agro-morphological, phenological and physiological data collected. A dendrogram was thus generated according for each type of temperature regime.

#### Relationship between the varieties under optimal condition

**Fig. 4:**
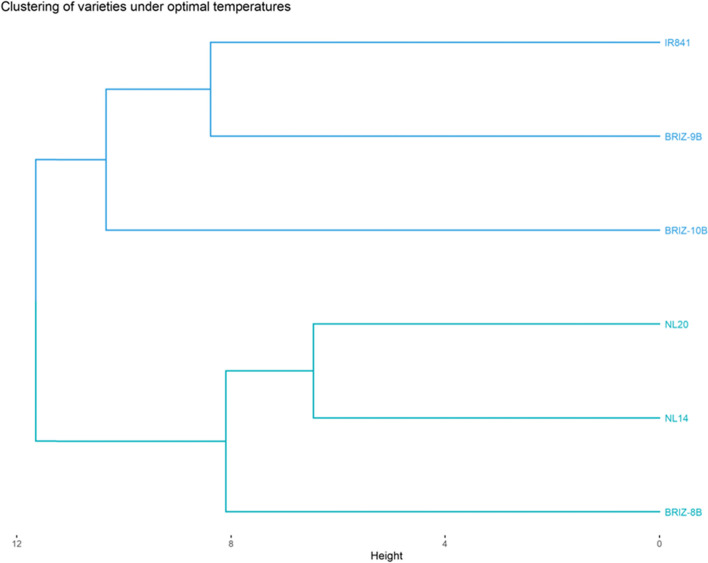
Clustering of the varieties under normal condition

The varieties were clustered into two classes and each cluster is made up of three varieties (Fig. 4). The first group consisted of BRIZ-8B NERICA-L14 and NERICA-L20 while the second group included BRIZ-10B, BRIZ-9B and IR841. Group 1 had the tallest plants, the biggest diameter at the base point of the stem or tiller, the longest and largest leaves, best vigorous plants, the longest internode, longest panicle, highest number of secondary branches, the highest days to maturity and the highest average yields per plant. The variety in group 2 longest grain. Finally, class 3 had varieties with a considerable number of tillers and internodes, longest flowering dates, and highest thousand grain weight compared to the other classes.

#### Relationship between the varieties under heat stress

**Fig. 5:**
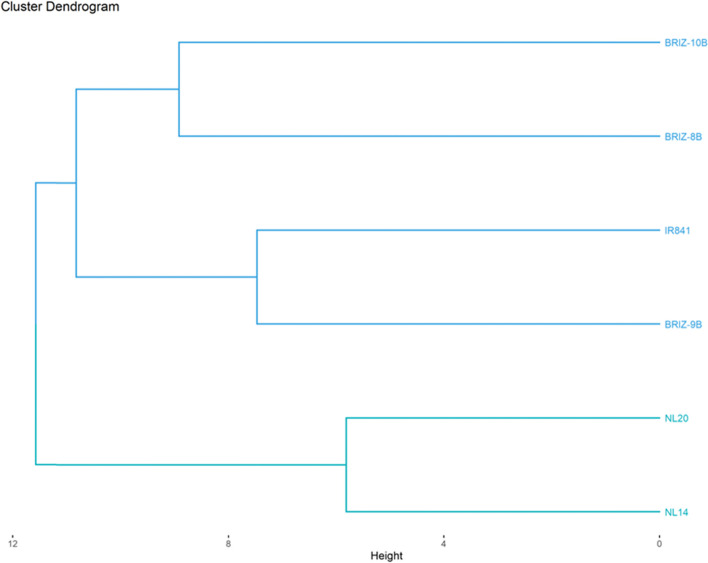
Clustering of the varieties under heat stress

The clustering analysis grouped the varieties into three classes (Fig. 5). Class 1 consists of two varieties BRIZ-10B and BRIZ -8B. Class 2 is also made up of two varieties BRIZ-9B and IR841. Finally, the last class is constituted by the varieties NERICA-L14 and NERICA-L20. Individuals in class 1 showed the tallest plants, longest leaf, leaf width at 56 days after transplanting, biggest diameter at the base point of the stem or tiller, longest internode, the highest number of internodes, best vigorous plants, longest panicle leaf width, the shortest days to 50% flowering, the lowest Fo30, and Fm30, the highest Fv/Fm at 40 days and as well as the highest weight of 1000 grains. The varieties of class 2 had the shortest plant height, the largest number of tillers and internodes, the shortest panicle length, the highest values of initial and variable fluorescence, the highest ratio between the initial and variable fluorescence. As for the varieties in class 3 they had the smallest diameter at 30 days after transplanting and number of internode at 30 days, the lowest number of tillers at 30 days, longest panicle leaves, highest value of initial fluorescence, longest panicles, highest number of secondary branches, longest days to 50% flowering and maturity, and longest grains with the highest yields per plant.

### Estimation of genetic parameters

#### Phenotypic and genotypic variation

Phenotypic variance was greater than genotypic variance for all variables under both normal and heat stress conditions. Phenotypic variance ranged from 0.03 to 297.30 under optimal conditions and from 0.01 to 193.30 under heat stress. The plant height at 56 days after transplanting (297.30 optimal conditions and 193.30 under stress) shows the highest phenotypic variance. This variance is followed by panicle leaf length (99.77 under optimal and 72.77 under stress), leaf length at 56 days (70.29 under stress), and grain maturity date (61.27 optimal conditions). Similarly, genotypic variance ranged from 0.07 to 165 under optimal conditions and from 0.01 to 166.36 under heat stress. The traits with the highest genotypic variances were plant height at 56 days (165.03 optimal and 166.36 under stress), length of panicle leaves (85.67 optimal), days to 1% flowering (75, 26 under stress), and grain maturity date (55, 29 optimal).

#### Coefficient of phenotypic and genotypic variation

For all the traits, the coefficient of phenotypic variation was higher than the coefficient of genotypic variation irrespective of the season (Tables 6 and 7). Under optimal temperature conditions, the number of tillers at 56 days, the length of panicle leaves (23.86%), the average number of full grains (28.43%), the average number of empty grains (55.05%), the average yield per plant (25.62%), and the yield per plot (46.40%) had the highest phenotypic variation coefficients. Under heat stress conditions, plant height at 30 days and 56 days (20.18%), diameter at the base point of the stem or tiller measured at 56 days (30.51%), number of internodes at 30 days (23.80%) and 56 days (27.85%), the length of internodes at 30 days (21.02%), the length of panicle leaves (23, 79%), number of panicles (25.39%), number of secondary branches (24.87%), the average number of empty grains (54.01%), average yield per plant (99.13%), yield per plot (46.40%) which had the highest phenotypic variation coefficient. The characters with moderate phenotypic variation coefficient under optimal conditions are plant height, leaf length measured at 30 days and 56 days after transplanting, number of tillers at 56 days, plant vigor at 30 days, panicle leaf length, thousand seed weight, and average yield per plant. Under heat stress, leaf length at 30 and 56 days, leaf width, diameter at 30 days, number of tillers, number of primary branches, and average number of filled grains had moderate phenotypic variation coefficients. On the other hand, the coefficients of low genotypic variation in optimal conditions are for leaf length at 56 days (9.52%), number of tillers at 30 days (7.85%), number of internodes, number of panicles (1, 98%), panicle length (3.91%), number of primary (2.67%) and secondary branches (7.64), kernel length (5.92%) and kernel width (2.45%), days to 50% flowering (6.94), and days to seed maturity (6.29%). While under heat stress the characters with the lowest genotypic coefficient of variation were leaf width at 56 days (7.97%), diameter at 30 days (6.42), number of tillers at 30 days and 56 days, internode length (8.99), panicle length (7.77), panicle length (7.35%), maturity date (6.29%). Under optimal temperature conditions, panicle leaf length (22.5%), average number of filled (22.73%) and empty grain (30.24), yield per plot (31.85%), had the highest genotypic variation coefficient. While under heat stress, diameter at 56 days (28.75), number of tillers at 30 (20.28) and 56 days (20.76), the length of internodes (24.51%), the average number of empty grain (41.56%), the average yield per plant (40.05%) and the yield per plot (36.54%) are those with the highest genotypic coefficient of variation**.**

**Table 6:** Genetic parameters of growth variables

Variables	Condition	VG	VP	H^2^(%)	GCV(%)	GCP(%)
HP30	Optimal	38.33	48.83	78.51	11.99	13.54
	Stress	38.36	52.87	72.55	13.75	20.18
HP56	Optimal	165.03	297.3	55.51	14.68	19.71
	Stress	166.36	193.3	86.06	18.72	20.18
LargF30	Optimal	0.007	0.01	70	10.86	12.62
	Stress	0.01	0.02	50	13.22	15.87
LargF56	Optimal	0.013	0.018	72.22	9.52	11.43
	Stress	0.009	0.017	52.94	7.97	11.28
Diat30	Optimal	1.54	3.11	49.51	11.1	15.76
	Stress	0.16	1.25	12.8	6.42	17.54
Diat56	Optimal	5.07	13.23	38.32	9.62	15.53
	Stress	51.14	57.59	88.79	28.75	30.51
NbT30	Optimal	0.53	2.64	20.05	7.85	17.53
	Stress	0.09	0.68	13.23	6.49	17.65
NbT56	Optimal	1.87	3.5	56.17	15.51	20.69
	Stress	0.17	2.35	7.23	2.86	11.01
NbEN30	Optimal	0.004	0.2	2	1.71	12.82
	Stress	0.28	0.4	70	20.28	23.8
NbEN56	Optimal	0.08	1.51	5.62	2.5	10.54
	Stress	5.55	9.95	55.74	20.79	27.85
LongEN30	Optimal	0.06	0.16	37.5	8.99	14.05
	Stress	0.09	0.14	64.28	17.75	21.02
LongEN56	Optimal	0.46	1.28	35.93	9.61	16.05
	Stress	1.19	2.05	58.06	24.51	32.17
LongFP	Optimal	85.69	99.77	85.89	22.05	23.79
	Stress	7.66	72.76	10.53	7.77	23.86
LargFP	Optimal	0.08	0.17	47.05	13.19	18.52
	Stress	0.1	0.17	58.74	18.39	24

**Table 7:** Genetic parameters of performance variables

Variables	Condition	GV	PV	H^2^(%)	GCV(%)	GCP(%)
NPa	Optimal	0.04	1.81	2.2	1.98	13.24
	Stress	2.04	5.3	38.48	15.77	25.39
LongPa	Optimal	3.87	5.28	73.17	7.35	8.59
	Stress	1.05	3.78	27	3.91	7.43
NbRFP	Optimal	0.07	1.81	3.86	2.67	13.24
	Stress	0.79	1.38	57.35	9.38	12.38
NbRFS	Optimal	4.2	5.72	73.46	7.64	8.91
	Stress	6.9	14.26	48.38	17.31	24.87
NbMGP	Optimal	58.83	91.8	63.96	22.73	28.43
	Stress	51.66	158.66	32.52	7.65	13.42
NbMGV	Optirmal	11.97	39.67	30.17	30.24	55.05
	Stress	30.52	45.08	67.71	44.44	54.01
LongG	Optimal	0.32	0.34	93.67	5.92	6.12
	Stress	0.051	0.207	24.75	2.37	4.76
LargG	Optimal	0.002	0.005	49.13	2.45	3.5
	Stress	0.03	0.033	92.72	8.75	9.09
DatF1%	Optimal	75.26	90.29	83.34	10.1	11.06
	Stress	53.7	78.39	68.5	7.72	9.33
DatF50%	Optimal	42.06	46.4	90.66	6.94	7.29
	Stress	11.26	36.43	30.92	3.14	5.65
DatM	Optimal	55.29	61.27	85.34	6.29	6.81
	Stress	15.67	22.6	69.33	3.05	3.67
P1000G	Optimal	25.4	26.7	95.11	15.9	16.31
	Stress	2.32	13.64	17.04	5.17	12.54
RdmtPlt	Optimal	0.008	0.02	34.03	14.62	25.62
	Stress	0.05	0.27	18.88	43.05	99.13
RdtPa	Optimal	0.92	1.35	78.34	31.85	38.53
	Stress	1.2	2.21	54.55	36.54	46.4

#### Broad sense heritability of the traits

Under normal temperature conditions, the variables with the highest heritability were leaf length at 56 days (97.98%), thousand grain weight (95.11%), and days to 50% flowering (90.66%) panicle leaf length (85.89%), grain maturity date (85.34%), leaf length at 30 days (84.21%), days to 1% flowering (83.34), plant height at 30 days (78.98%), and panicle length (73.17%) (Tables 6 and 7). Under heat stress, heritability was higher for the following traits: plant diameter at the base point of the stem or tiller (88.79%), leaf length at 56 days (87.04%), plant height at 56 days (86.06%), height at 30 days (72.55%), number of internodes at 56 days (70.00%), days to 50% flowering (69.33%), days to 1% flowering (68.50%).

## Discussion

### Growth parameters under heat stress

The results of the analysis of variance showed that the temperature regimes and the variety influenced plant height 30 days after transplanting, the length of the plant, and the length of internodes 56 days after transplanting. The tallest plants under optimal conditions of temperatures and heat stress are BRIZ-8B, NERICA-L14, and NERICA-L20. In fact, these varieties are all interspecific. BRIZ-8B is obtained by crossing a line of TOG5681 with IR64. NERICA-L14 and NERICA-L20 are interspecific with *O. glaberrima* and *O. sativa* [22]. According to [23] the *O. glaberrima* and TOG5681 lines are identified as tolerant to heat stress based on plant size and chlorophyll content. BRIZ-9B was more sensitive to heat stress due to its short plant height observed under both temperature conditions. This is consistent with the work of [24, 25] who demonstrated that high temperature influenced negatively the growth parameters of rice plants. At the same time, BRIZ-10B shows lower height variation when subjected to both temperature conditions. The longest leaves observed in BRIZ-8B under heat stress shows the level of tolerance of this variety to high temperature. The shortest leaves were observed in BRIZ-9B indicating its sensibility to heat stress [26]. showed that an increase in temperature can decrease the rate of seed germination, as well as cause growth inhibition and early senescence of leaves. A temperature higher than 36 °C was observed during the growth phase of heat-stressed plants. It can therefore be stated that this increase in temperature above 36 °C during the growth phase would have had negative effects on the various growth parameters measured. This confirms the results of [27] who showed that temperatures above 32 °C negatively affect all stages of growth and development of rice plants.

### Effect of heat stress on yields and yield attributes

The results showed that temperature affected rice yield and its components, notably the days to flowering, days to grain maturity, number of secondary branches, the weight of a thousand grains, and the number of empty grains per panicle.

Heat stress affects the days to flowering of different rice varieties. The shortest day to flowering date was observed in the variety BRIZ-10B, under both optimal and heat stress conditions. We observed that there was an increase in days to flowering under heat stress compared to the optimal temperature condition for all the varieties studied, which is in line with previous results [20, 28]. Under heat stress, during flowering a temperature above 36 °C is recorded. This increase in temperature is therefore responsible for the prolonged days to flowering observed in all varieties. This confirms the results of [11] who showed that above 33 °C, the flowering time of rice plants is delayed. High temperature leads to the modification of days to flowering of rice plants, subsequently causing sterility of spikelet [29]. Flowering (anthers and fertilization) is the most sensitive developmental stage to high temperature in rice [19] and this was observed in the different varieties used in the present study. Variability in flowering and grain maturity dates were much more observed in IR841, NERICA-L14, and NERICA-L20. The Lowest variation was noticed in BRIZ-10B and BRIZ -8B. It indicates that these two varieties BRIZ-10B and BRIZ-8B are tolerant to high temperatures based on days to flowering and grain maturity.

The high temperatures also influenced the thousand grain weight of the different rice varieties. Low thousand grain weights of the varieties were observed under heat stress compared to those obtained at normal temperature. Under high temperature, the post-harvest weight of rice grains decreases considerably [30]. Under heat stress, BRIZ-10B recorded the lowest thousand grain weight. At the same time, BRIZ-8B and NERICA-L14 showed the highest thousand grain weights. Our results are in agreement with [25, 31, 32]. Due to heat stress during the period of maturity, rice grain filling was affected [33]. High temperatures of more than 32 °C during flowering result in reduced grain yield [18]. Heat stress at the onset of the flowering stage reduced the thousand grain weight of rice. The probable reason could be due to pollen sterility or inhibition and the growth of spikelet or poor grain filling [34]. The weight of thousand grains is mainly determined by the development of the integument and endosperm [34]. In adverse environments, such as high temperature, hull size, and grain weight decreased due to abnormal spikelet development [34]. In addition, the growth and development of the rice plant are also influenced by the high temperature at the initial stage of reproduction, which can have negative effects on grain filling [34]. Therefore, this explains the low thousand-grain weights in different varieties under heat stress.

The early rainfall that occurred after 50% flowering was beneficial to the recovery of plants from heat stress. However, the activity of heat stress at the time of grain filling is at the origin of the loss of weight obtained at the end compared to that obtained in optimal conditions.

There was a negative correlation between the high temperature and days to flowering, the weight of thousand grains. The correlation on these variables also confirms the effect of heat stress on flowering and yield. These results corroborate the work of [35] who showed that heat stress affects reproductive development and grain yield of rice.

### Effect of heat stress on chlorophyll fluorescence

The results showed that heat stress had no significant influence on the physiological parameters studied. This is contrary to the work of [36, 37] who indicated that drought-induced heat stress influences the physiological mechanisms of rice plants. The very low influence of heat stress on the physiology of rice plants observed could be explained by the non-destruction of the photosynthetic apparatus of the 6 varieties by the long-term mild heat recorded during the trial. This allowed the production and distribution of biomass, especially in the reproductive organs, thus contributing to the different yields obtained. However, the work deserves to be deepened at this level in order to evaluate the capacity of physiological adaptation of these various varieties under conditions of heat stress.

The current study has shown that there could be a combination of plant height, flowering date, and thousand seed weight and heat stress tolerance for some varieties (BRIZ-8B, NERCA-L14, and N BRIZ-10B). The varieties BRIZ-8B, NERICA-L14, and BRIZ-10B developed some mechanisms of tolerance to high temperature, since these varieties demonstrated the highest weight of thousand grains compared to those of the other varieties. In addition, they showed fewer empty spikelets compared to the other varieties.

The results of ANOVA also showed that temperature regime and variety factor significantly affected the number of secondary branches and the maturity date of grains.

PCA results showed that the highest thousand seed weight was associated with higher yield per plant and plot and that a variety with high thousand seed weight under heat stress or normal temperature indicated the highest yield per plant or plot. This is because good grain filling at the time of heat stress resulted in good yield [34].

### Genetic variability

Genetic parameters show that genotypic variation is lower than phenotypic variation in all traits except for height, grain length, and thousand seed weights under heat stress. These results are in agreement with [38]. Low genotypic and phenotypic variance reflects low impact of environmental factors on trait expression; while high variance shows the impact of environmental factors on traits. This finding is consistent with previous reports on rice [39]. According to [40], the coefficients of phenotypic variation are low below 11%, moderate between 11 and 20%, and high above 20%. The coefficient of phenotypic variation was higher than the coefficient of genotypic variation for all traits. But the significant difference in some characters such as height, diameter at the base point of the stem or tiller, number of thalli, length of panicle leaves, number of panicles, 1000 seed weight, average yield per plant, and per plot showed that these factors were influenced by the environment under heat stress. The highest PCV % and GCV % values will allow the improvement of genotypes for high temperature tolerance through selection for these desired traits. On the other hand, the lower values of PCV % and GCV % such as panicle length, grain length and width, flowering date, maturity date, indicate the lack of variability; which limits the improvement through the selection of these traits. Our results are in agreement with [41–43].

Low, medium and high values of broad heritability were obtained for the different traits. The low values under heat stress were for diameter at the base point of the stem or tiller, internode length, thallus number, panicle leaf length, panicle number, average number of filled grains per panicle and thousand grain weight, suggesting that it will be difficult to improve these traits. These results are in contrast with those obtained by [44–46] on rice, which can be explained here by the genetic and environmental difference. High heritability was observed with plant height, flowering date, internode length, panicle leaf width, number of primary branches and yield per plot; suggesting that these traits are highly heritable. The heritability value alone gives no indication of the amount of genetic advance that could result from the selection of the best individuals [47]. Expected genetic advance as a percentage of means revealed variable behavior of traits. In this study leaf length showed high heritability value coupled with a high genetic advance value indicating that genetic effects of additive type are important in determining this trait. The number of primary branches, flowering date and yield per plot showed high heritability coupled with a high genetic advance indicates the effective chance of selection of these traits for yield improvement. Our results are similar to [48–50].

## Conclusion

In conclusion, the six varieties studied responded differently under heat stress conditions. Our results showed that the BRIZ-8B variety is the most tolerant variety to heat stress on the basis of the morphological and agronomic traits measured. Also, the variety BRIZ-10B is tolerant to heat stress based on yield traits. These varieties (BRIZ-8B, and BRIZ-10B) could serve as breeder’s starting material or recommended to farmers for cultivation under heat stress conditions. This study also revealed great morphological and agronomic variability in rice plants. It was noted that there is an influence of environmental factors on the expression of traits under heat stress, illustrated by a more or less large gap between the genotypic and phenotypic coefficients of variation and a high heritability in the broad sense for most of the traits studied.

## Supplementary Information

**Additional file 1.**

## Data Availability

The datasets used and analyzed during the current study are available from the corresponding author on reasonable request.

## Abbreviations

GV: Genotypic Variance
PV: Phenotypic Variance
H^2^: Heritability in the broad sense
GCV: Coefficient of genotypic variation
PCV: Coefficient of phenotypic variation
EG: Expected genetic gain
HP30: height at 30 days after transplanting (in cm)
HP56: height at 56 days after transplanting (in cm)
Diat30: diameter at 30 days after transplanting (in mm)
Diat56: diameter at 56 days after transplanting (in mm)
LongF30: leaf length at 30 days after transplanting (in cm)
LongF56: leaf length at 56 days after transplanting (in cm)
NbT30: number of tillers at 30 days after transplanting
NbT56: number of tillers at 56 days after transplanting
NbEN30: number of internodes at 30 days after transplanting
NbEN56: number of internodes at 56 days after transplanting
LongEN30: internode length at 30 days after transplanting (in cm)
LongEN56: internode length at 56 days after transplanting (in cm)
LongFP: panicle leaf length (in cm)
LargFP: panicle leaf width (in cm)
NPa: number of Panicle
LongPa: panicle length
DatF1%: date of flowering at 1% plant
DatF50%: date of flowering at 50% plant
DatM: date at grain maturity
LongG: grain length (in mm)
LargG: grain width (in mm)
NbRFP: number of primary branching
NbRFS: number of secondary ramification
NbMGP: average number of full grains per panicle
NbMGV: average number of empty grains per panicle
P1000G: weight of thousand grains
RdmPlt: average yield per rice plant (in kg/m2)
RdPa: yield per plot (in t/ha)
Fo30: Initial value of fluorescence after 30 days of transplanting
Fo40: Initial value of fluorescence after 40 days of transplanting
Fm30: Maximum value of fluorescence after 30 days of transplanting
Fm40: Maximum value of fluorescence after 40 days of transplanting
Fv30: Fluorescence to variable taken at 30 days after transplanting
(Fv/F0)30: Ratio between variable and initial fluorescence taken at 30 days after transplanting
(Fv/F0)40: Ratio between variable and initial fluorescence taken at 40 days after transplanting
(Fv/Fm) 30: Ratio between variable and maximum fluorescence taken at 30 days after transplanting
